# Bullous Pemphigoid Development in a Patient Treated with Dulaglutide: A Case Report

**DOI:** 10.51894/001c.164571

**Published:** 2026-07-31

**Authors:** Natalia Baj-Osiewicz, Noora Khadoori, Isabella Gluzman

**Affiliations:** 1 Henry Ford - Genesys

## 63

### Introduction

Bullous pemphigoid (BP) is a rare autoimmune skin disorder with an annual incidence ranging from 2.4-21.7 per million population worldwide. The development of BP has been associated with various medications including antidiabetic medications, although rarely with glucagon like peptide 1 (GLP-1) receptor agonists. Here we describe the 5th case in literature that we know of a GLP-1 agonist-induced BP.

### Case Presentation

An 83-year-old White female with a past medical history of insulin-dependent type II diabetes mellitus complicated by peripheral neuropathy, chronic kidney disease type IIIa and hypothyroidism was engaged in routine primary care follow up for management of diabetes. The patient was taking high doses of long- and short-acting insulins, and empagliflozin with inadequate glucose control. Therefore, the decision was made to start dulaglutide at the lowest dose (0.75 mg weekly). The patient did not have any contraindications to dulaglutide. She remained on this starting dose for 8 weeks. The dose was then increased to 1.5 mg weekly, as she was tolerating the medication well without any side effects. The patient was referred to a dermatologist for an evaluation of multiple facial seborrheic keratoses. Patient noted a skin blister on the right forearm which was approximately three months after the initiation of dulaglutide. The lesion was described as a tense bulla with surrounding erythema. Two 4mm punch biopsies were obtained by the dermatologist. The patient was treated with clobetasol 0.05% twice daily. Two weeks later, the rash expanded to include bilateral anterior forearms, and body surface area 1%. Biopsy results showed a linear deposition of IgG, IgG4 and C3 along the basement membrane pattern consistent with pemphigoid, ulceration with underlying perivascular lymphoid infiltrative with eosinophils consistent with ulcerated bullous pemphigoid. Dulaglutide was discontinued. Skin lesions resolved with the use of clobetasol without any recurrence.

### Conclusion

Despite the significant clinical benefits of GLP-1 agonists, it is crucial to beware of rare but serious medication side effects. We highlight the importance of detecting skin lesions in concomitance with GLP-1 use even weeks later. Here, we present the 5th case in literature that we are aware of, of GLP1-induced bullous pemphigoid.

